# Experiences of families of adolescents with gender incongruence in the light of the Calgary Models for Families

**DOI:** 10.1590/1980-220X-REEUSP-2022-0027en

**Published:** 2022-07-18

**Authors:** Paula Fernanda Lopes, Luciana de Lione Melo, Circéa Amália Ribeiro, Vanessa Pellegrino Toledo

**Affiliations:** 1 Universidade Estadual de Campinas, Campinas, SP, Brazil.; 2 Universidade Federal de São Paulo, São Paulo, SP, Brazil.

**Keywords:** Office Nursing, Gender Identity, Adolescent, Family, Case Reports, Mental Health, Enfermería de Consulta, Identidad de Género, Adolescente, Familia, Informes de Casos, Salud Mental, Enfermagem no Consultório, Identidade de Gênero, Adolescente, Família, Relatos de Casos, Saúde Mental

## Abstract

**Objective:**

to know the experiences of family members of adolescents with gender incongruence.

**Method:**

this is a qualitative case study, supported by the Calgary Family Assessment and Intervention Models theoretical-methodological framework. Data collection took place through semi-structured interviews, participant observation in family groups and document analysis, with eight family members. Data analysis was performed following the precepts of content analysis.

**Results:**

with family assessment, two categories emerged: “Challenges in the face of gender transition”, which highlighted the problems related to the expectations created at birth, new names, pronouns and gender fluidity and the fear of prejudice, and “Supporting aspects in the face of the possibility of gender transition”, which revealed family support as a strong point.

**Conclusion:**

knowing the experiences allowed us to understand the challenges that family members face when facing physical and emotional aspects of their children’s gender transition. It was noticed that the act of seeking help and offering support is important for a healthy transition. The findings provided a better understanding of family issues and provided suggestions on how nursing can develop care for this population.

## RESUMO

**Objetivo:**

conhecer as experiências de familiares de adolescentes com incongruência de gênero.

**Método:**

estudo de caso qualitativo, apoiado pelo referencial teórico-metodológico dos Modelos Calgary de Avaliação e Intervenção Familiar. A coleta dos dados ocorreu por meio de entrevistas semiestruturadas, observação participante em grupos de familiares e análise documental, com oito familiares. A análise dos dados foi realizada seguindo os preceitos da análise de conteúdo.

**Resultados:**

com a avaliação familiar, emergiram duas categorias: “Desafios em face à transição de gênero”, que evidenciou os problemas relacionados às expectativas criadas ao nascimento, novos nomes, pronomes e fluidez de gênero e o medo do preconceito, e “Aspectos apoiadores diante da possibilidade de transição de gênero”, que revelou o apoio familiar como ponto forte.

**Conclusão:**

conhecer as experiências permitiu compreender os desafios que os familiares enfrentam ao se deparar com aspectos físicos e emocionais da transição de gênero de seus filhos. Percebeu-se que o ato de procurar ajuda e de oferecer apoio é importante para uma transição saudável. Os achados proporcionaram melhor compreensão das questões familiares e forneceram sugestões de como a enfermagem pode elaborar o cuidado a essa população.

## RESUMEN

**Objetivo:**

conocer las vivencias de familiares de adolescentes con incongruencia de género.

**Método:**

estudio de caso cualitativo, sustentado en el referencial teórico-metodológico de los Modelos de Evaluación e Intervención Familiar de Calgary. La recolección de datos se llevó a cabo a través de entrevistas semiestructuradas, observación participante en grupos familiares y análisis de documentos, con ocho familiares. El análisis de los datos se realizó siguiendo los preceptos del análisis de contenido.

**Resultados:**

con el diagnóstico familiar surgieron dos categorías: “Desafíos frente a la transición de género”, que destacó los problemas relacionados con las expectativas creadas al nacer, nuevos nombres, pronombres y fluidez de género y miedo al prejuicio, y “Aspectos de apoyo ante la posibilidad de transición de género”, que revelaron el apoyo familiar como punto fuerte.

**Conclusión:**

conocer las experiencias permitió comprender los desafíos que enfrentan los miembros de la familia al enfrentar aspectos físicos y emocionales de la transición de género de sus hijos. Se notó que el acto de buscar ayuda y ofrecer apoyo es importante para una transición saludable. Los hallazgos proporcionaron una mejor comprensión de los problemas familiares y proporcionaron sugerencias sobre cómo la enfermería puede desarrollar el cuidado de esta población.

## INTRODUCTION

The way people express themselves in the world and recognize themselves concerns their identity. When gender identities or behaviors are not congruent with a person’s assigned sex at birth, the term “gender incongruence” is used. The prevalence of children and adolescents with gender incongruence is difficult to determine; it is estimated to correspond from 0.3% to 1.2% of the population^(1,2)^.

A small part of these children can develop, in adolescence, significant suffering, caused by this discrepancy between the gender identity and the sex assigned to the person at birth (and the associated gender role and/or primary and secondary sexual characteristics), termed as gender dysphoria^(3)^.

Interventions that may be beneficial for individuals with gender incongruence include psychotherapy, gender social transition, hormone therapy, gender-affirming surgery, and/or other follow-up services. Considering the adolescent population, transition begins with social transition^(4)^.

Social transitions in children and adolescents involve changing pronouns, hairstyle, clothing and sometimes name to align with gender identity, which differs from that designated at birth. Unfortunately, by violating gender expectations rooted in the culture of society, those who differ from the pre-established become the target of discrimination, constituting a marginalized and vulnerable community, prone to experience more health and psychosocial problems than other groups^(5)^.

The existing literature shows that gender transition is a family process and thus affects all its members in various forms and intensities^(6,7)^. It is necessary to understand that the parents of these adolescents can develop feelings of loss, grief and uncertainties, given the exposure of a gender identity different from that experienced since birth^(8–10)^. Thus, nurses who consider the importance of families may find ways to develop interventions that promote a healthy environment^(6–10)^.

In this context, families have sought professional help, but find professionals who are not prepared to welcome and identify their needs^(11)^. According to a survey of transgender people who sought a health service, a third of them reported negative experiences related to their gender identity, involving neglect of their social name, verbal harassment and refusal of care^(11)^. The lack of knowledge to develop nursing care can also result in guidance to families without foundations and that often expose them to situations of judgments and prejudices by those who should act in the opposite direction^(12)^.

Favoring a supportive family environment is directly related to greater satisfaction with life and, consequently, fewer depressive symptoms in adolescents with gender incongruence. Thus, optimizing parental support and understanding their challenges is essential, as they provide nursing professionals and other members of the health team with a foundation to develop care aimed at understanding the needs and health disparities of this population^(5–13)^.

However, considering the extensive literature affirming the importance of families in the development of a healthy gender transition, there is a lack of research that presents a look focused on their needs, especially on the experiences of parents of having a child who is experiencing a series of physical and emotional changes, which also affect them^(1–13)^. Similarly, when thinking about nursing care for this population, there is an advance in studies specifically directed to transgender individuals, but these, generally, do not go into depth in the attention to their surroundings, justifying the development of research that provides references that support practice^(5,6)^.

Thus, this study aimed to know the experiences of family members of adolescents with gender incongruence.

## METHOD

### Design of Study

This is a single and incorporated qualitative case study^(14)^, which follows the Consolidated criteria for Reporting Qualitative research (COREQ) recommendations^(15)^.

### Participants

Five mothers and three fathers of five adolescents in gender transition attended in a gender and sexuality outpatient clinic participated, totaling eight family members.

### Local

Gender and sexuality outpatient clinic of a teaching hospital in the countryside of the state of São Paulo. The service offers multidisciplinary care to children and adolescents aged four to 20 years who experience incongruence with their birth sex. It aims to assess and promote the mental health and quality of life of this population, as well as that of their families, through outpatient follow-up by health professionals, such as psychiatrists, endocrinologists, gynecologists, nurses, psychologists, speech therapists, art therapists, among others.

### Selection Criteria

Family members, close to adolescents (identified by the construction of a genogram), being over 18 years of age and being available to attend nursing consultations and family groups were included. People with non-daily living or proximity to adolescents were excluded.

### Sample Definition

Selection was made based on initial contacts with families, choosing to select those that were more frequent in the outpatient’s activities, which were identified by the researcher and the other professionals in the team, to ensure continuity in the research.

### Data Collection

Three major methods of data collection are considered in the case study: asking questions, observing events and reading documents^(14,16)^. The sources of evidence used in this study were participant observation, semi-structured interview and documentary analysis. In its three forms, data collection was carried out in the months between February 2019 and February 2020, monthly, on the days scheduled for family groups, i.e., with a duration of ten days (considering that there were no meetings in July and December due to the outpatient’s recess), which resulted in approximately 40 hours of observation.

Data collection began through semi-structured interviews, conducted with the elected members of each family in outpatient clinics and recorded by an audio device, after their opinion, lasting about 30 minutes. As a script, a pre-elaborated instrument was used based on Calgary Family Assessment and Intervention Models (CFAM/CFIM). The instrument proposed presenting families by questions referring to the three main categories of CFAM/CFIM: structural, developmental and functional^(13)^.

The structural category aims to understand the family structure and the relationship between its members, data obtained from the construction of a genogram and ecomap. The development category refers to the progressive transformation of family history, accessed by questions such as: what were the greatest changes in raising this adolescent? What is happening in their lives? The functional category refers to how family members interact. Thus, questions such as: how do you realize that your child is dealing with the situation? How is the family handling the^(13)^ situation?

In a second moment, parents were invited to participate in the family group, which resulted in total compliance, favoring the development of participant observation not only through informal conversations in the outpatient clinic’s corridors.

In each meeting with the group, the leaders (researcher and a psychologist from the outpatient clinic team) introduced themselves, explained the group’s objectives, which consisted of offering a safe environment for the exchange of experiences between people who are going through a common situation, emphasizing its importance as a source of data for this study. Family members were invited to introduce themselves, as well as their children, and were encouraged to tell the reason for their inclusion in the group. In all sessions, this movement was sufficient to raise a theme to be discussed and, thus, the meetings developed. Records were made immediately in a field diary, in order to avoid the loss of relevant information from the observed data, as recommended in the literature^(17,18)^.

Medical records were accessed in order to identify the reason why family members had sought the outpatient clinic, which contained the changes perceived in adolescents, related to their gender identity.

### Data Analysis and Treatment

The transcripts of the interviews and the field diary records were organized according to the content analysis framework, which covers three stages with the purpose of signifying the collected data: pre-analysis, material exploration and treatment of results, through inference and interpretation^(19)^.

It started by rereading the interviews and field diary records, aiming to identify the relevant aspects referring to the context of families’ experience; identification and subsequent grouping of significant aspects of statements into units of meaning; synthesis of meaning units to later compose the thematic categories to be discussed^(19)^.

The data obtained from the medical records were organized in a table, in order to present family members who sought the service, the age of their child and the first changes in gender expression perceived.

The reports and observations from data collection, which refer to problems faced by participants, were grouped in the category “Challenges in the face of gender transition”, which understands the difficulties presented by family members when experiencing the gender transition of one of their members. Moreover, throughout the meetings, patterns of strengths were identified, presented in the category “Supporting aspects in the face of the possibility of gender transition”, which represent the perceived strengths, characterized as the support that the family offers and its ability to accept receiving help.

### Ethical Aspects

The study was approved by the Research Ethics Committee of UNICAMP, under Opinion 3,049,342, in 2018, and followed all the recommendations of Resolution 466/2012 of the Brazilian National Health Council. Data collection began after participants’ consent through the signing of the Informed Consent Form and the Assent Form for voice recording. To preserve participants’ anonymity, adolescents’ names were replaced by the colors Orange, Blue, Green, Yellow and Violet, referring to the LGBTQIA + pride flag.

## RESULTS

Each family participated in at least one consultation for assessment. In the chart below, data related to family assessment of adolescents with gender incongruence are related, presenting the history obtained through the interview and data from adolescents’ medical records.

**Chart 1.**
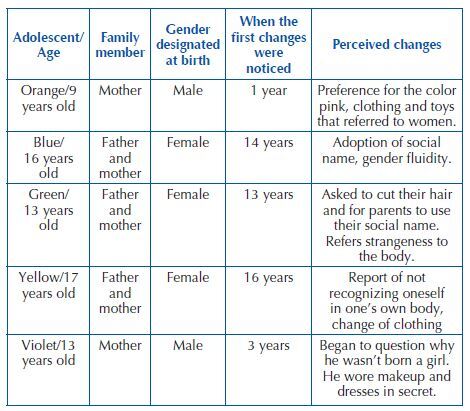
History of adolescents with gender incongruence according to participating family – Campinas, SP, Brasil, 2022.

### Gender Transition Challenges

Family members’ experiences express their anguish regarding the changes arising from the gender transition of their children. Adapting images constructed and referring to gender of birth were recurring subjects in all meetings.

*I remember when she was born, I held her, I saw her genitals, I saw a vagina, it was really a girl (…) then you already think of names and what you think it will be like when you grow up. (Yellow’s Father)*

*I’m going to wake him up, kiss him and say, “Good morning, princess”, as I always have.* (Green’s Father)

*I ended up changing the way I get her attention, because it’s not the same way I talk like my oldest son. With him, I get excited, I scream, but with Orange I try to be more delicate.* (Orange’s Mother)

Embarrassments continue when they make a mistake when using the new names and pronouns in dialogues with the person in transition. Sometimes they are censored and, other times, they are themselves who identify their own slips and correct themselves.

*The sister manages to call her in the male, but we don’t. There are times when I get careless, and she, he, complains. (Blue’s Father)*

*You have to call him in front of grandma “she”. Then my other son gets confused, because he’s used to calling “him”.* (Green’s Mother)

Moments of meeting with other family members afflict parents, who feel pressured to share or not the identity of their children.

*The father wanted to tell the family at first. And I said no! Because she wasn’t ready. But now I’m waiting for her to be ready, but I don’t think that’s going to happen. (Blue’s Mother)*

*I want to tell, not to stand on the fence! Because we’ve been in this situation for months.* (Blue’s Father)

Beyond family borders, there are public environments. Family members suffer for thinking about what can happen to their children in a prejudiced society that may not accept this discordant identity and even cause violent actions, especially when they start to change their look, such as their haircut and clothes.

*At school she goes as a “he”. As she has been there since the first year, now is in the fourth year, it is very difficult for you to sleep boy and wake a girl. So, even for her, this is very difficult. There are barriers and prejudices and she has full conviction of this. (Orange’s Mother)*

*I get scared because, you saw how it is, right? The clothes show the body, the curves* (…) *so I’m afraid of how they can see her and want to take advantage.* (Violet’s Mother)

Gender fluidity, perceived in everyday actions, related to its expression, such as clothing, use of makeup, haircut, makes parents cling to the hope that the child may change his mind and not continue with the social transition.

*This week he was tidying up his wardrobe, sorting out all the girl’s clothes to donate. Then I went there to see how she was doing and I saw that she had really taken off everything, except for two dresses that she really liked and the music box, then it gave me that hope, right! (…) I don’t know if he’s already made up his mind or not. I wish it were more decisive. (Blue’s Mother)*

At that moment, the mother searched for a photo on her cell phone to show the group. Yellow’s mother, with teary eyes, shows a photo of her son, now a trans boy, wearing a bikini on the beach, and extols his feminine characteristics.

*Today, for example, you can see outside, if you put on makeup, you’re wearing earrings. We don’t understand! But I don’t think it’s bad either, no. (Green’s Father)*

### Supporting Aspects Faced with the Possibility of Gender Transition

Considering the problems that family members face, all of them demonstrated the ability to provide support, security and encouragement to adolescents, i.e., supportive aspects in the face of the possibility of gender transition. The fact of looking for a specific outpatient clinic for gender issues, with a focus on mental health, encourages this finding.

*I go to her rhythm! Oh, is that the problem? So, let’s go after that. She gives me options… so we go after what she says. I take her shopping for clothes because she likes a certain style, but she chooses. (Orange’s Mother)*

*She doesn’t want to go to college right now… so we talked to the psychologist about giving her credit, reliable… so we support him to study at home. See if he can plan and study at home…* (Blue’s Mother)

*We come to groups, come to consultations, so we can really understand and be with her in decisions, because she still depends on us, she’s under 18 years old, so we go together, we participate…* (Green’s mother)

*I don’t know if he’s already made up his mind or not… I wish it were more decisive… but we’ll support what he decides.* (Blue’s Father)

Just as they demonstrate their ability to support their children, family members understand that they also need help in this process.

*We [mother and boyfriend] talk about everything and especially about that and for me it is a support, something that does not happen with her father! (Orange’s Mother)*

*At these times, you realize who really matters in your life, and if you don’t want to be a part, that’s fine, you can go.* (Yellow’s Mother)

Other parents say they are in follow-up with psychology professionals and that the indication occurred through the care of their children.

*I’m in therapy and it’s been really good for me, you know. (Blue’s Mother)*

*After I started to understand from the side of the family constellation my way of thinking changed and it is being very important!* (…) *before I faced how to grieve, my daughter died and now I have a son. Now I understand that it remains the same person, only with different name and clothes… is a light being, like an engineering… what happened was a reconstruction, not death.* (Yellow’s Mother)

They also extol the importance of the support received in the meetings at the gender outpatient clinic, listening to stories similar to their own, exchanging information, with the support of a multidisciplinary team.

*We’ve heard a lot of stories here, in the group, that’s cool! (Blue’s Father)*

*This exchange that happens here, I think super important!* (Violet’s Mother)

In the chart below, the problems and strengths listed from the meetings are summarized, as well as the proposed interventions:

**Chart 2.**
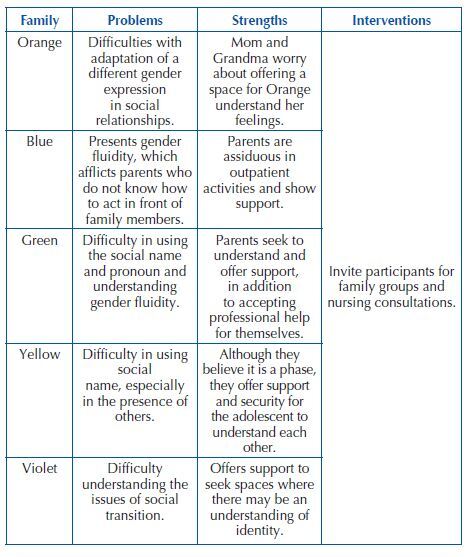
Problems, strengths and interventions of participating families – Campinas, SP, Brasil, 2022.

## DISCUSSION

Based on the knowledge of experiences of families of adolescents with gender incongruence through the application of CFAM/CFIM, it was possible to identify issues that are characterized as problems and strengths related to the experience of gender transition of one of the members.

The problems were present at various levels of the family system. At the structural level, for instance, in the matter of adjusting to the new family form, with a child going through a social transition, adapting to a gender incongruous to the one assigned at birth. At the level of development, in relation to the family’s life cycle and in the affective bonds between its members. And at the functional level, in the belief in the heterocisnormative pattern that needs to be reconstructed, in the expression and demonstration of feelings^(13)^.

Participants brought up the difficulty in dealing with the steps of adolescents’ gender transition, defined as a generic term for the steps that a trans person and their community take to affirm their gender identity. Depending on age and individual needs, these steps may include social, medical, surgical, and legal changes. In adolescents, gender transition is mainly a social process^(4,13)^.

Gender transition simultaneously affects the individual and family system, influencing members to varying degrees. The greatest challenge in the face of change in one person is that it will not be possible for others to respond as they used to, and for parents this can mean abandoning their dreams so that their children can live theirs. Moving from someone who cared for a daughter to someone who now cares for a child, for instance, implies creating new meanings and perceptions of this relationship, which may require that those original expectations for the adolescents’ future be abandoned^(4,6,13)^.

Parents relate the choice of name to a period long before birth, in which they already planned and already wondered what their children would look like. Studies suggest that parents of children belonging to sexual minorities may be suffering from the loss of an image nurtured by them since the birth of their child that becomes evident when this adolescent begins to publicly manifest their gender identity in a way that does not correspond to their biological sex, through their name, clothing, haircut, behavior, voice and/or body characteristics and how they interact with the other people^(20,21)^.

Amidst the process of developing adjustments to changes in both the individual and the family system, parents may also deal with conflicting feelings of loyalty between the decisions made by their children and society’s expectations. Corroborating the results of a previous study, participants in this study were unsure of how to act or what to say to other people as the transition progressed, in an attempt to simultaneously address their own feelings and those of their children^(6)^.

It is common for families to feel isolated in the process of understanding these feelings, reinforcing the importance of offering safe environments that welcome these people and provide for the development of trusting relationships, in which it is possible to identify ways to promote adjustment to problems in family functioning^(6)^.

From the family assessment, it was observed that family groups consisted of few people, usually only by parents and children. In order to expand the support network, the present study suggested the participation of parents in support groups offered by the outpatient clinic itself as an intervention, corroborating findings from previous studies^(6,7,13,22)^. Participation in support groups where people who are facing similar problems are brought together encourages family members to tell their own narratives, which encourages them to deal with the situations faced^(7,13)^.

As understanding and acceptance develops, parents may continue to experience loss and sadness, helplessness about the situation, and anxieties about the future, including whether their child will truly remain transgender^(6,7)^. This doubt is manifested in most participating families, when they perceive a fluidity in adolescents’ identity and/or gender expression. In this context, nurses can intervene by accessing the cognitive domain, offering information on issues that permeate gender identity^(13)^.

Gender fluidity refers to the temporal change in a person’s expression or gender identity or both. This change can be in the expression, but not in identity, or identity, but not in the expression, or both can change together^(22,23)^. For some young people, fluidity can be a way to explore gender before reaching a more stable gender expression or identity. For others, gender fluidity can continue indefinitely as part of their life experience with gender^(22,23)^.

As in a previous study, participants showed urgency in witnessing the outcome of the gender transition process, evidenced by the discomfort in seeing a child living “between genders”^(7)^. This tension generated in the family system can result in a scenario in which, on the one hand, parents live in a state of anticipation when the expected result is apparently prolonged, and, on the other hand, they are unaware of or do not conceive of the possibility of adolescents presenting a non-binary gender identity or one that remains fluid^(7)^.

Recognizing their anguish, all participants in this study reported seeking professional help, either by a professional or by a trusted person, which was listed as a strong point to be explored. Family members of adolescents with gender incongruence need to be heard and share their stories and often turn to support groups that favor the construction of the meaning of that situation in their lives, which corroborates the findings presented^(6,7,9,24)^.

By promoting opportunities for family members to express their narratives, CFAM/CFIM propose to empower them to extract their own strengths and resources for mutual support. Nurses can be the catalyst that will facilitate communication between family members or between them and other health professionals^(6,13)^. For this, using interventional questions can be an option to reach the affective family domain, seeking to understand which members are suffering, how they are behaving and thus knowing their perceptions and reactions about a given problem^(13)^.

Validating this intense affection can alleviate feelings of isolation and loneliness and help family members make the connection between the situation experienced by one of them and the emotional response of all the others. As recommended by CFAM/CFIM, it is important that nurses validate these emotions, encourage the narrative and support^(6,7,13)^.

Family support for adolescents with gender incongruence has been associated in literature with greater satisfaction with life and social relationships and fewer depressive symptoms^(6–9)^. Likewise, lack of support constitutes a risk of family rejection, suicide, discrimination and reluctance to seek health services^(6–8)^.

Adolescents with gender incongruence are considered one of the most marginalized and oppressed school groups. Some studies suggest that sexual minorities are gaining greater visibility and, consequently, greater acceptance. However, students with gender incongruence continue to face a more hostile school climate compared to their homosexual or bisexual peers, for instance^(25)^.

Family assessment is generally taught at the undergraduate nursing level; however, the limited focus on family nursing theory and gender identity issues may be insufficient to develop the knowledge and skills needed to care for this population, which is a limitation of its application^(13)^.

Thus, knowing these experiences should impel nursing to think about a care that emphasizes the importance of family role, not in the development of gender incongruence, nor in its cure, but in their ability to find collaborative ways to nurture their children, to support their personality and choices. These measures are essential to establish limits, ensure safety and prepare to make informed decisions about the potential medical and social transition or lack thereof^(6,13,26)^.

## CONCLUSION

The methodological approach of the qualitative case study contributed to understanding the experiences of families of adolescents with gender incongruence, revealed through CFAM/CFIM in an outpatient gender and sexualities in childhood and adolescence.

The scope of experiences using an assessment and intervention model allowed the knowledge that these family members go through a series of challenges when accompanying an adolescent in a gender transition. While progress has been made in caring for transgender individuals and in recognizing their rights, the specific focus on this population does not seem to offer the same space for those with whom they live to have their needs heard.

CFAM/CFIM proved to be effective tools in this sense by providing nurses with involvement with these families, in order to assess, explore and identify strengths and problems and make decisions to intervene. The difficulties that family members face when faced with physical and emotional aspects of their children’s gender transition need to be welcomed and managed by nurses, based both on knowledge of their peculiarities and on a methodology that supports their practice.

It was noticed that family members have strengths, evidenced by the intention to seek help and offer support to adolescents, but it is up to nursing to facilitate access to health services and advocate for their rights. Developing a relationship of mutual trust and empathy and favoring the narrative of these families to express their feelings and problems can enable them to use their strengths to create tools for overcoming difficulties.

Finally, it is pointed out that this study presented as a limitation the fact that it was a case cut from a single outpatient clinic, comprising only the families studied, which opens possibilities for the development of new studies.
